# Rational Function-Based Approach for Integrating Tableting Reduced-Order Models with Upstream Unit Operations: Dry Granulation Case Study

**DOI:** 10.3390/ph17091158

**Published:** 2024-08-31

**Authors:** Sunidhi Bachawala, Rexonni B. Lagare, Abigail B. Delaney, Zoltan K. Nagy, Gintaras V. Reklaitis, Marcial Gonzalez

**Affiliations:** 1 School of Mechanical Engineering, Purdue University, West Lafayette, IN 47907, USA; sbachawa@purdue.edu; 2 Davidson School of Chemical Engineering, Purdue University, West Lafayette, IN 47907, USA; 3 Ray W. Herrick Laboratories, Purdue University, West Lafayette, IN 47907, USA

**Keywords:** dry granulation, tableting, reduced-order models, bivariate rational functions, granule size distribution, ribbon density

## Abstract

We present a systematic and automatic approach for integrating tableting reduced-order models with upstream unit operations. The approach not only identifies the upstream critical material attributes and process parameters that describe the coupling to the first order and, possibly, the second order, but it also selects the mathematical form of such coupling and estimates its parameters. Specifically, we propose that the coupling can be generally described by normalized bivariate rational functions. We demonstrate this approach for dry granulation, a unit operation commonly used to enhance the flowability of pharmaceutical powders by increasing granule size distribution, which, inevitably, negatively impacts tabletability by reducing the particle porosity and imparting plastic work. Granules of different densities and size distributions are made with a 10% *w*/*w* acetaminophen and 90% *w*/*w* microcrystalline cellulose formulation, and tablets with a wide range of relative densities are fabricated. This approach is based on product and process understanding, and, in turn, it is not only essential to enabling the end-to-end integration, control, and optimization of dry granulation and tableting processes, but it also offers insight into the granule properties that have a dominant effect on each of the four stages of powder compaction, namely die filling, compaction, unloading, and ejection.

## 1. Introduction

The pharmaceutical industry is transitioning from batch manufacturing to continuous manufacturing. The Quality by Design (QbD) framework guidelines, introduced by the Food and Drug Administration (FDA) in 2004, have been instrumental in redefining the pharmaceutical industry’s approach to product development and manufacturing. QbD ensures product quality by design rather than by inspection, thereby mitigating risks and reducing the likelihood of defects or manufacturing deviations [1,2]. Complementing the QbD paradigm is the concept of Quality by Control (QbC), which underscores the importance of real-time monitoring and control of critical process parameters in continuous manufacturing [3,4].

**Figure 1:**
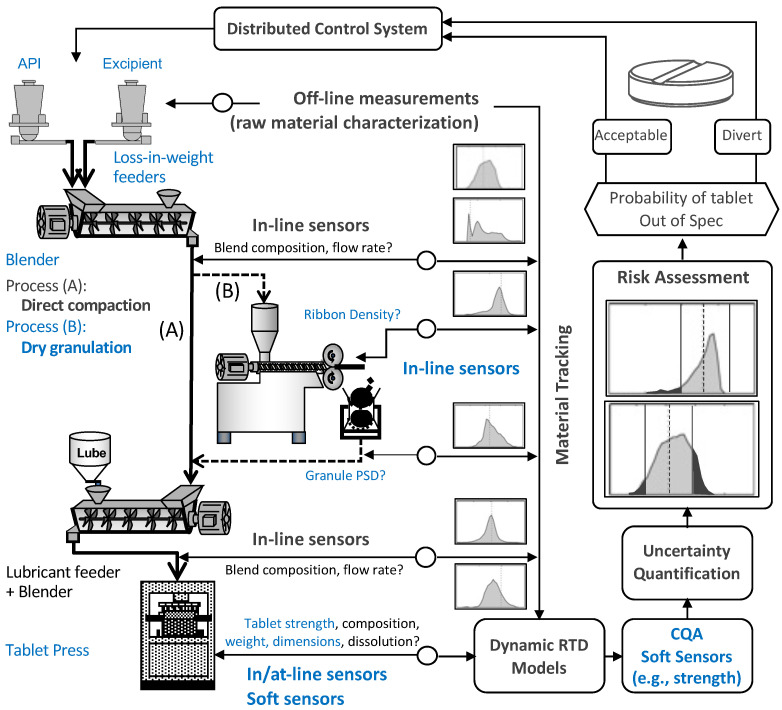
The continuous solids-processing pilot plant at Purdue University involves several steps: (i) K-Tron and Schenck feeders are used to feed the APIs and excipients, respectively, ensuring a uniform blend in a Gericke GCM-250 blender; (ii) based on the manufacturing process (direct compaction or dry granulation), the powder blend is mixed with a lubricant feed from the K-Tron MT12 micro-screw lubricant feeder or fed to an Alexanderwerk WP120 roller compactor for dry granulation; and (iii) the final step involves the production of solid tablets using a Natoli NP-400 rotary tablet press.

By way of illustration, Figure 1 shows the continuous solids-processing pilot plant situated at Purdue University. Within this experimental setup, the Active Pharmaceutical Ingredients (APIs) and the excipients are fed through K-Tron and Schenck feeders, respectively. The powders are then homogeneously mixed in a Gericke GCM-250 continuous blender. Following this step, the choice between direct compaction or dry granulation dictates the path for the powdered blend, involving the utilization of a K-Tron MT12 micro-screw lubricant feeder or an Alexanderwerk WP120 roller compactor. Finally, the production of solid tablets is achieved by utilizing a Natoli NP-400 rotary tablet press. In continuous manufacturing, the variations in the upstream unit operations have a cascading effect on the downstream unit operations. Particularly in the dry granulation manufacturing route, changing the roller compactor settings will affect the granule properties, eventually affecting the downstream tableting critical process parameters (CPPs) and tablet critical quality attributes (CQAs). Here, for simplicity, the tablet CQAs are weight, tensile strength, and tablet relative density. The CPPs are the process variables that directly impact the tablet CQAs, such as the compaction force and in-die relative density of the tablet.

Real-time monitoring and control of unit operations require accurate models to predict intermediate product properties and tablet CQAs. Tableting models under the direct compaction route in Figure 1 have been extensively studied [5,6,7]. Semi-mechanistic models used to capture the effects of lubricant or glidant feeding on tablet weight, strength, and density have been proposed by Bachawala and Gonzalez [6]. Furthermore, Ferdoush and Gonzalez [7] proposed semi-mechanistic reduced-order models (ROMs) to predict changes in the dissolution profile due to process disturbances in tablet weight, porosity, and lubrication conditions. These nonlinear mechanistic models were further used to develop and implement nonlinear model predictive control (NMPC) to control a tablet press in silico [4] and were demonstrated in the physical pilot plant [8] using the Natoli NP-400 rotary tablet press.

In the dry granulation–tableting line illustrated in Figure 1, the roller compactor converts powder to granules, which are then passed into the tablet press to form tablets. In the roller compactor, powder is first compressed to a ribbon, which is then milled to form granules. Dry granulation improves blend uniformity and flowability due to the enlargement of granules. The downside is that the tabletability of the granules is significantly reduced due to the work hardening the granules during roll compaction [9]. Therefore, as tableting mechanistic ROMs have been instrumental in implementing NMPC for direct compaction, developing mechanistic models to predict tablet CQAs for the dry granulation and tableting line is of paramount importance.

Existing mechanistic models for roller compaction [10,11] and direct compaction [6,12] are typically independent of each other. However, when the roller compactor and tablet press are used simultaneously, the tablet CQAs directly correlate with granule properties. For example, the bulk density of the powder blend must be replaced with the bulk density of the granules, among other considerations [6]. There have been successful attempts to predict tablet CQAs based on roller compactor CPPs, such as roll force and screw speed, using regression models [13,14]. However, regression models are not translatable to other equipment or formulations.

In this work, we present a systematic and automatic approach for integrating tableting ROMs with upstream unit operations that not only identifies the upstream critical material attributes (CMAs) and CPPs that describe the first- and, possibly, second-order coupling, but also selects the mathematical form of such coupling and estimates its parameters. Specifically, we propose that the coupling can be generally described by normalized bivariate rational functions. This approach is illustrated by integrating tableting ROMs with dry granulation CMAs and CPPs. To this end, a design of experiments is carried out to capture the effect of roller compactor CPPs (such as roll force and gap) and CMAs (such as ribbon relative density) on the tableting process. Effects on compaction force and the tablet’s elastic recovery, density, and tensile strength are studied, and model parameters are estimated. Granules with 10% acetaminophen and 90% microcrystalline cellulose are fabricated using an Alexanderwerk WP120 roller compactor, and tablets are fabricated using a Natoli NP-400 tablet press. It is worth noting that a salient aspect of the proposed approach is that it can be extended and applied to any other roller compactor, tablet press, or product formulation. Lastly, these ROMs are essential in realizing the end-to-end integration, control, and optimization of dry granulation and tableting processes, beyond the scope of this work.

The rest of this paper is organized as follows. Section 2 outlines the experimental preparation and characterization of granules and tablets used in this study. Section 3 presents the mechanistic and semi-mechanistic tableting ROMs adopted for each of the four stages of powder compaction, namely die filling, compaction, unloading, and ejection. Section 4 introduces an approach to couple these tableting ROMs with upstream unit operations systematically and to automatically select the mathematical form of such coupling, i.e., of the normalized bivariate rational function, and estimate its parameters. Section 5 shows how effective this approach is in building ROMs that integrate dry granulation and tableting processes. The mathematical form of the optimal models selected, their parameters, and the mechanistic insights that emerge from them are presented in Section 6. Conclusions and remarks are addressed in Section 7. Finally, future work and areas for improvement are discussed in Section 8.

## 2. Materials and Methods

### 2.1. Preparation and Characterization of Granules

The formulation was composed of 90% by mass of microcrystalline cellulose Avicel PH-102 (MCC), from IMCD Group US, and 10% by mass of acetaminophen Grade 0048 (APAP), from Mallinckrodt Pharmaceuticals. Lubricants and glidants, such as magnesium stearate and colloidal silica, were not used. In turn, the true density of the formulation $\rho_{t}$, as measured with the Accupyc II 1340 pycnometer, was 1.558 g/cc. Six different batches of granules were prepared by employing an Alexanderwerks WP120 roller compactor with varying roll pressures (namely 30, 60, and 90 bar) and roll gaps (namely 2 and 3 mm).

Three-kilogram batches with the mixture of 10% APAP and 90% MCC were first pre-blended in a five-liter Tote blender for 30 min. A Schenck Accurate feeder was used to feed the pre-blend into a Gericke GCM-250 continuous blender, operating at 150 rpm, and next into the roller compactor. The powder blend was compressed into ribbons as the granulator feedscrew pushed powder through two counter-rotating rolls. Next, a two-stage hammer mill with two screens, namely 2.5 and 1.25 mm, crushed the ribbons into granules. The hydraulic roll pressure and the roll gap are control variables for which setpoints can be varied continuously by virtue of two control modules, referred to as *feedback gap control* and *pressure control*. The operating range of the Alexanderwerks WP120 roller compactor is 1–4 mm for the roll gap and 0–230 bar for the roll pressure. In this study, the roller compactor was operated for two minutes after startup to reach steady-state conditions, the roll and mill speeds were kept constant at 4 and 25 rpm, respectively, and granules were collected at the output of the roller compactor for characterization purposes and for making tablets in the rotary tablet press.

To model the effect of roller compactor settings on CQAs, we first mapped the ribbon and granule properties (CMAs) of the in-process material, i.e., the granules, with the roll pressure, roll gap, roll speed, mill speed, and screen size values (CPPs). Specifically, the model developed by Huang et al. [15] was used to estimate the ribbon relative density $\rho^{\mathrm{ribbon}}$ and granule size distributions (GSDs) of each batch. This hybrid model combines Johanson’s model [16] for roll compaction with a machine learning (ML) model to maintain high physical interpretability and feasibility. The model accounts for elastic recovery of the ribbon to estimate the ribbon relative density $\rho^{\mathrm{ribbon}}$, i.e., the ratio of the apparent density of the ribbon to the true density of the blend $\rho_{t}$. The GSD produced by the roller compactor hammer mill was approximated using a bimodal Weibull distribution, as follows:

(1) $$P\left(x\right)=\alpha \left[\frac{k_{1}}{\lambda_{1}}{\left(\frac{x}{\lambda_{1}}\right)}^{k_{1}-1}e^{-{\left(\frac{x}{\lambda_{1}}\right)}^{k_{1}}}\right]+\left(1-\alpha \right)\left[\frac{k_{2}}{\lambda_{2}}{\left(\frac{x}{\lambda_{2}}\right)}^{k_{2}-1}e^{-{\left(\frac{x}{\lambda_{2}}\right)}^{k_{2}}}\right],$$

where α is the fraction corresponding to the small mode, $\lambda_{1}$ and $k_{1}$ are the size and shape parameters of the small mode, and $\lambda_{2}$ and $k_{2}$ are those of the large mode. Hence, the mean granule size $\mu^{\mathrm{GSD}}$ is obtained from the mean size of each component of the bimodal Weibull distribution (i.e., $\mu_{1}$ and $\mu_{2}$) as

(2) $$\mu^{\mathrm{GSD}}=\alpha \mu_{1}+\left(1-\alpha \right)\mu_{2},$$

with

$$\mu_{1}=\lambda_{1}\Gamma \left(1+1/k_{1}\right)$$

$$\mu_{2}=\lambda_{2}\Gamma \left(1+1/k_{2}\right),$$

where Γ(·) is the gamma function, defined as $\Gamma \left(x\right)=\int_{0}^{\infty }s^{x-1}e^{-s}ds$.

**Figure 2:**
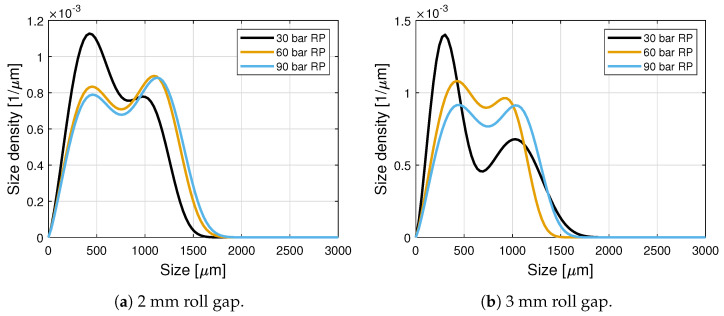
Granule size distributions for different roll pressure and roll gap processing conditions estimated using the hybrid model of Huang et al. [15].

The hybrid model was calibrated by Huang et al. [15] using experimental data for the same formulation used in this work, under processing conditions similar to those used in this work. Table 1 lists the hybrid model parameters, and Figure 2a,b show the estimated GSDs for 30, 60, and 90 bar of roll pressure and 2 and 3 mm of roll gap. Lower roll pressures result in lower ribbon densities, and a higher percentage of fines. It is worth noting that a 1.5 mm roll gap produces a GSD very similar to the one obtained using a 2 mm roll gap.

It is worth noting that these CMAs can be measured off-line, at-line, and in-line. The ribbon envelope density can be measured off-line using the GeoPyc 1360 pycnometer. The GSD of granule samples can be measured using the Canty SolidSizer, which measures the size and area of each particle [15]. The ribbon relative density and particle size can be measured in-line using near-infrared (NIR) monitoring [17,18,19]. The Innopharma Eyecon2, which uses a high-speed imaging technique, has also been used to measure the granule size distribution in real time [20].

### 2.2. Preparation and Characterization of Tablets

**Figure 3:**
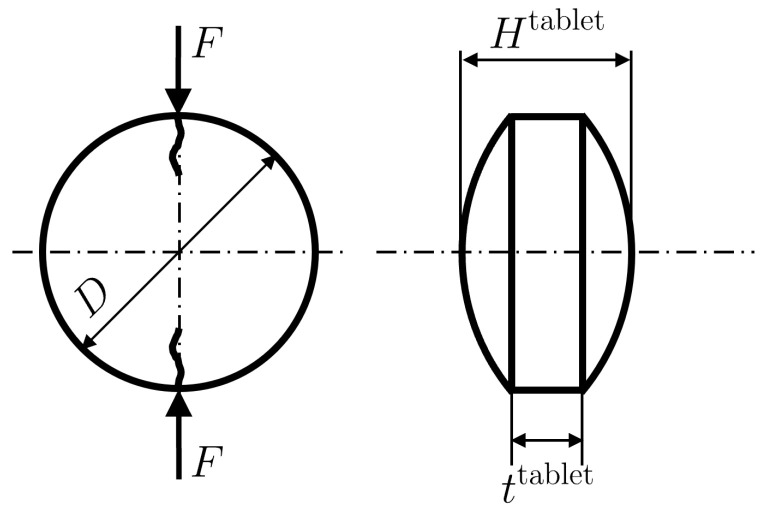
Diametrical compression of doubly convex tablets.

The granules produced by the roller compactor were further used to make tablets. Tablets were manufactured in a Natoli NP-400 tablet press using a doubly convex D-type tool with a standard cup depth of h=0.3302 mm and a diameter of D=7.94 mm, as illustrated in  Figure 3. The turret speed, feed-frame speed, and pre-compression thickness were kept constant at 25 rpm, 40 rpm, and 5 mm, respectively. For each of the six batches of granules generated with the roller compactor, we made tablets of four different relative densities. In this section, we first describe the steps taken to create the design of experiments, and subsequently, we describe the tableting process and how product data are acquired.

The operating ranges of control variables in the tablet press are (i) 6–14 mm for the dosing position, (ii) 1–5 mm for the pre-compression and main compression thickness, and (iii) 5–60 rpm for the turret and feed-frame speed. The maximum punch tip force is 50 kN. However, it is challenging to choose dosing position and main compression thickness values that create tablets of the desired relative density within the operating limit of the punch force. It requires the knowledge of prior experimental data, which is not always available. Here, we used the tableting models established by Bachawala et al. [6] for direct compaction of the same formulation to guide the estimation of dosing position and main compression thickness values conducive to safe operation conditions. Specifically, the objective was to target a broad range of tablet relative densities, namely from 0.6 to 0.95, while not exceeding the maximum tool force of the punches. Given a target relative density, the bulk density of the granules is needed to, in turn, guide the estimation of the dosing position and main compression thickness. Hence, a 500 mg sample of each batch was divided into eight sub-samples using a Retsch PT100 spin riffler, and the weight and volume of each sub-sample were measured. Similarly, given a target relative density, the compaction force needed for direct compaction [6] was used to guide the estimation of an upper limit. Once these limits were established, we proceeded to make tablets with the least and highest relative densities. In most cases, tablet strength was developed at the lowest relative density value, and the limit of the punch tip force was not exceeded at the highest one. Had we identified that any of these limits were incorrect, we would have increased/decreased the dosing position in increments of 0.5 mm, until safe operation conditions were established. Following this procedure, the main compression and dosing position limits were chosen for each batch of granules, as shown in Table 2.

As shown in Figure 1, the equipment used to manufacture pharmaceutical tablets at Purdue’s continuous manufacturing pilot plant is lined up vertically with provisions for in-line, at-line, and off-line measurements of CQAs and CMAs. For each tableting run, a SOTAX AT4 tablet tester was used to measure the thickness, diameter, weight, and hardness of 100 tablets under steady-state manufacturing conditions. Tablet press control variables and critical process parameters were measured and collected in real time by the Delta-V data historian. Therefore, the pre-compression thickness $t^{\mathrm{pc}}$, in-die main compression thickness $t^{\mathrm{in}-\mathrm{die}}$, pre-compression force $F_{\mathrm{pc}}$, and main compression force $F_{\mathrm{punch}}$ values for each run are stored in the Delta-V data historian. At-line measurements for the weight *W*, thickness $H^{\mathrm{tablet}}$, diameter *D*, and hardness *F* of doubly convex tablets (Figure 3) were obtained from the SOTAX AT4 tablet tester. Tablet CQAs, tablet density, and tensile strength were further calculated from the SOTAX AT4 measurements.

During the die-filling process, the fill depth or dosing position $t^{\mathrm{fill}}$ is met by positioning the bottom convex punch, while the scraper wipes off the excessive powder. Next, the upper punch compresses the powder bed at the pre-compression and the main compression stages using preset thicknesses, i.e., $t^{\mathrm{pc}}$ and $t^{\mathrm{in}-\mathrm{die}}$, respectively. Upon unloading and ejection, the tablet relaxes elastically to a tablet thickness $t^{\mathrm{tablet}}$. The corresponding tablet volumes are given by

(3) $$V^{\mathrm{fill}}=\frac{\pi D^{2}t^{\mathrm{fill}}}{4}+\frac{\pi h}{12}\left(\frac{3D^{2}}{4}+h^{2}\right)$$

(4) $$V^{\mathrm{pc}}=\frac{\pi D^{2}t^{\mathrm{pc}}}{4}+\frac{\pi h}{6}\left(\frac{3D^{2}}{4}+h^{2}\right)$$

(5) $$V^{\mathrm{in}-\mathrm{die}}=\frac{\pi D^{2}t^{\mathrm{in}-\mathrm{die}}}{4}+\frac{\pi h}{6}\left(\frac{3D^{2}}{4}+h^{2}\right)$$

(6) $$V^{\mathrm{tablet}}=\frac{\pi D^{2}t^{\mathrm{tablet}}}{4}+\frac{\pi h}{6}\left(\frac{3D^{2}}{4}+h^{2}\right),$$

with $t^{\mathrm{tablet}}=H^{\mathrm{tablet}}-2h$ (see Figure 3). For a given filling weight *W*, the corresponding relative densities are given by

(7) $$\rho^{\mathrm{pc}|\mathrm{in}-\mathrm{die}|\mathrm{tablet}}=\frac{W}{\rho_{t}\;V^{\mathrm{pc}|\mathrm{in}-\mathrm{die}|\mathrm{tablet}}}.$$

Lastly, the tensile strength $\sigma_{t}$ for convex tablets is obtained from Pitt’s model [21] as recommended by Razavi et al. [12] using the measured hardness *F*, i.e.,

(8) $$\sigma_{t}=\frac{10F}{\pi D^{2}\left[2.84\left(H^{\mathrm{tablet}}/D\right)0.126\left(H^{\mathrm{tablet}}/t^{\mathrm{tablet}}\right)+3.15\left(t^{\mathrm{tablet}}/D\right)+0.01\right]}.$$

It bears emphasis that not all measurements are available on-line to implement real-time control under the QbC paradigm [4]. Therefore, one needs predictive models to estimate the CMAs and CQAs, which are available at-line or off-line [6,7]. These models can then be deployed to control the equipment and the process in real time under the QbC framework, if beyond the scope of this paper. Tableting reduced-order models that predict the tablet CQAs are described in the next section.

## 3. Tableting Reduced-Order Models

**Figure 4:**
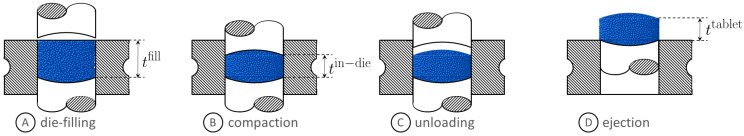
Stages of tablet compaction. Image courtesy of [5].

The development of mechanistic and semi-mechanistic reduced-order models (ROMs), resulting from a trade-off between complexity and performance but still based on product and process understanding, forms an essential cornerstone for process design, optimization, and control in pharmaceutical manufacturing. Tableting ROMs have been developed for each of the four stages of compaction (see Figure 4). For ideal filling conditions, the tablet weight *W* can be estimated from the bulk density $\rho_{b}$ (measured off-line) and the fill volume of the die $V_{\mathrm{fill}}$, i.e., from $W=\rho_{b}V_{\mathrm{fill}}$. In the rotary tablet press, however, the efficacy of the filling process depends on the flowability of the powder blend and the turret and feed-frame paddle speeds. In addition, the bulk density might not be equal to the one measured off-line, due to segregation and mixing taking place inside the feed frame [22]. These two effects are lumped into a filling efficacy coefficient η, defined as follows:

(9) $$\eta =\frac{W}{\rho_{b}V_{\mathrm{fill}}}.$$

During the main compaction stage, the tablet is compacted to the desired thickness, and, consequently, the desired in-die tablet relative density $\rho^{\mathrm{in}-\mathrm{die}}$. The compaction force $F_{\mathrm{punch}}$ model proposed by Kawakita and Lüdde [23], which is based on powder compression mechanisms, has been modified by Bachawala and Gonzalez [6] to depend on the critical in-die relative density $\rho_{c}$ at which jamming occurs and particle-level deformations begin [6]. The model has the following form:

(10) $$F_{\mathrm{punch}}=\frac{\pi D^{2}\left(\rho^{\mathrm{in}-\mathrm{die}}-\rho_{c}\right)}{4b\left(\rho^{\mathrm{in}-\mathrm{die}}\left(a-1\right)+\rho_{c}\right)},$$

where the parameters *a*, *b* [1/MPa] are model parameters related to material properties. During the unloading stage, the tablet expands and undergoes elastic recovery. The model found by Gonzalez [5] for elastic recovery $\epsilon_{\rho }$ is generalized to

(11) $$\epsilon_{\rho }=\epsilon_{0}{\left(\frac{\rho^{\mathrm{in}}-\mathrm{die}-\rho_{c,\epsilon }}{1-\rho_{c,\epsilon }}\right)}^{n},$$

where $\epsilon_{0}$ is the in-die elastic recovery at full compaction (or zero porosity), $\rho_{c,\epsilon }$ is the smallest in-die tablet relative density at which elastic recovery is observed, and *n* is an exponent that accommodates for nonlinearities and generalizes the otherwise linear trend. Hence, the out-of-die tablet relative density is computed from the elastic recovery using

(12) $$\rho^{\mathrm{tablet}}=\rho^{\mathrm{in}-\mathrm{die}}\left(1-\epsilon_{\rho }\right).$$

Lastly, the tensile strength of a tablet $\sigma_{t}$ is estimated using Leunberger’s model [24], i.e., by

(13) $$\sigma_{t}=\sigma_{0}\left[1-\left(\frac{1-\rho^{\mathrm{tablet}}}{1-\rho_{c,\sigma_{t}}}\right)e^{\left(\rho^{\mathrm{tablet}}-\rho_{c,\sigma_{t}}\right)}\right],$$

where $\rho^{\mathrm{tablet}}$ is the tablet relative density after elastic relaxation, $\sigma_{0}$ is the theoretical maximum tensile strength for a tablet relative density of one (or zero porosity), and $\rho_{c,\sigma_{t}}$ is the smallest tablet relative density at which a tablet with adequate strength is formed. The model is based on the concept of effective bonding contact points across the cross-sectional area of the compact and it assumes that the relative change in bonding points is proportional to the relative change in tablet density [24].

These semi-empirical or semi-mechanistic formulae are in remarkable agreement with calculations that use the particle mechanics approach for modeling the consolidation of powders under large deformations [5]. These three-dimensional mechanistic calculations utilize generalized loading–unloading contact laws for elasto-plastic spheres with bonding strength to describe inter-particle interactions, and thus they depend on two elastic, two plastic, and one fracture particle-level properties. The main focus of this work is to elucidate a systematic and automatic approach for integrating these tableting ROMs with dry granulation CMAs and CPPs.

## 4. Model Selection and Parameter Estimation

The systematic integration of tableting ROMs with upstream unit operations requires not only the identification of upstream CMAs and CPPs that describe the first- and, possibly, second-order coupling, but also to select the mathematical form of such coupling and estimate its parameters. We first propose that such coupling is solely through the model parameters.

ξ of the tableting reduced-order model M(ξ) by means of a nonlinear function of upstream CPP|CMA and its parameters θ, that is,

(14) M(ξ)∘ξ(CPP|CMA;θ)→M(CPP|CMA;θ)≡M(θ).

For example, for tablet tensile strength, $M\equiv \sigma_{t}$ and $\xi =\{\sigma_{0},\rho_{c,\sigma_{t}}\}$. Since upstream CMAs and CPPs have different units and order of magnitude, we next propose the following normalization and mapping to automate model selection and parameter estimation:

(15) $$\mathrm{CPP}|\mathrm{CMA}:x\in \left[\mathrm{lb},\mathrm{ub}\right]\to X={\left(\frac{x-\mathrm{lb}}{\mathrm{ub}-x}\right)}^{r_{X}}\in \left[0,+\infty \right),$$

where lb and ub are lower and upper bounds for the CPP|CMA*x*, and $r_{X}$ is a model parameter that accommodates for nonlinearities. For example, if the ribbon relative density is chosen as a CMA relevant to the coupling, then it has upper and lower bounds amenable to physical interpretation, i.e.,

$$\mathrm{CPP}|\mathrm{CMA}\equiv \rho^{\mathrm{ribbon}}\in \left(\rho_{c},\mathrm{rib},1\right),$$

where the lower bound is the smallest density or porosity at which a ribbon is formed and the upper bound corresponds to a ribbon of no porosity or a relative density of 1. Lastly, we propose that the coupling can be generally described by multivariate rational functions, which have been used to successfully interpolate and extrapolate sparse data in many applications [25,26]. Specifically, the following normalized bivariate rational function f(X,Y) is adopted:

(16) $$\xi \left(\mathrm{CPP}|\mathrm{CMA};\theta \right)\to f\left(X,Y\right)=\frac{p_{1}XY+p_{2}X+p_{3}Y+p_{4}}{q_{1}XY+q_{2}X+q_{3}Y+1},$$

such that $\theta =\{p_{i},q_{j},r_{X},r_{Y}\}$, with i=1,2,3,4,j=1,2,3, are the parameters of the coupling to be estimated, in general. This simple and versatile bivariate rational function has a limiting behavior that is amenable to physical interpretation, in as much as the upper and lower bounds identified for the CPP|CMA do. This limiting behavior is given by

(17) $$\begin{array}{cc} \; & f\left(0,0\right)=p_{4} \end{array}$$

(18) $$\begin{array}{cc} \; & f\left(0,+\infty \right)=p_{3}/q_{3} \end{array}$$

(19) $$\begin{array}{cc} \; & f(+\infty ,+\infty )=p1/q1 \end{array}$$

(20) $$\begin{array}{cc} \; & f\left(+\infty ,0\right)=p_{2}/q_{2}, \end{array}$$

and, for example, for the tensile strength model parameter $\sigma_{0}$, the functions

(21) $$\begin{array}{cc} \; & f\left(0,Y\right)=\left(p_{3}Y+p_{4}\right)/\left(q_{3}Y+1\right) \end{array}$$

(22) $$\begin{array}{cc} \; & f\left(+\infty ,Y\right)=\left(p_{1}Y+p_{2}\right)/\left(q_{1}Y+q_{2}\right) \end{array}$$

describe the dependency of the maximum tensile strength $\sigma_{0}$ of tablets made of granules whose relative density is equal to $\rho_{c,\mathrm{rib}}$ (i.e., X=0) and 1 (i.e., X→+∞), respectively, on a second CPP|CMA*y* mapped to *Y*, e.g., on some aspect of the GSD. The interpretability of some model parameters is a salient feature of the proposed approach. It is worth noting that these properties are retained if the rational function is simplified by equating to zero any subset of model parameters θ, which in turn adds versatility to the proposed approach for model selection and parameter estimation. By way of example, Figure 5 shows the case of CPP|CMA≡{x,y}∈[0.3,1]×[0,1]} and $\theta =\left\{p1,p2,p3,p4,q1,q2,q3,r_{X},r_{Y}\right\}=\left\{15,10,5,10,1,2,4,1,1\right\}$. Model selection and parameter identification then reduce to resolving a trade-off between model complexity, e.g., given by the number of model parameters $N_{p}$, and the goodness of the model predictions, e.g., given by the sum of squared errors (SSE) between model prediction M(θ) and experimental observation E. This is effectively quantified using the Akaike Information Criterion [27,28], defined as follows:

(23) $$\mathrm{AIC}=n\ln\left(\frac{\mathrm{SSE}}{n}\right)+2N_{p}=n\ln\left(\frac{1}{n}\sum_{i=1}^{n}{\left(M^{i}\left(\theta \right)-E^{i}\right)}^{2}\right)+2N_{p},$$

where *n* is the number of experimental data points. The model with the lowest AIC is the best. Therefore, a library of rational functions F can be built by progressively simplifying f(X,Y) for each of the two CPPs|CMAs adopted and, in turn, reducing the number of model parameters $N_{p}^{m}$ in $\theta^{m}$, for model *m* in the library. For example, if the tensile strength model parameter $\sigma_{0}$ is known to depend only on the ribbon density, then $p_{1}=p_{3}=q_{1}=q_{3}=0$ and is removed, together with $r_{Y}$, from θ. Therefore, the optimal model $\bar{m}$ and its corresponding parameters $\bar{\theta }$ are given by

(24) $$\left\{\bar{m},\bar{\theta }\right\}=\arg\min_{m\in F}\left[n\;\ln\left(\frac{1}{n}\min_{\theta^{m}\in D^{m}}\sum_{i=1}^{n}{\left(M^{i}\left(\theta^{m}\right)-E^{i}\right)}^{2}\right)+2N_{p}^{m}\right],$$

where $D^{m}$ are nonlinear inequality constraints for the accepted range of model parameters $\theta^{m}$. In this work, we use MATLAB’s genetic algorithm (ga) fmincon and global search (GlobalSearch) in that specific order to solve the constrained nonlinear optimization problem and obtain parameters. This ensures that the estimated parameters are not biased to the initial guess and represent the global solution. The identification of two CPPs|CMAs, their upper and lower bounds, and an appropriate model library F for integrating dry granulation and tableting processes is discussed next.

We close by noting that regardless of the units used for M and E in the estimation of SSE, the model with the lowest AIC value will be the same. Hence, for simplicity, units of SSE values reported hereafter are omitted.

## 5. Reduced-Order Models to Integrate Dry Granulation and Tableting Processes

The systematic integration of the tableting ROMs presented in Section 3 with dry granulation is carried out by following the model selection and parameter estimation approach proposed in Section 4. Firstly, the ribbon relative density $\rho^{\mathrm{ribbon}}$ and mean granule size $\mu^{\mathrm{GSD}}$ are identified as the two upstream CPPs|CMAs that describe to the first and second orders the coupling between unit operations. Next, upper and lower bounds for the ribbon relative density are set to be 1 (i.e., granules with no porosity) and $\rho_{c,\mathrm{rib}}$ (i.e., the smallest density or porosity at which a ribbon is formed). Forming a ribbon is equivalent to forming a direct compression tablet. Therefore, $\rho_{c,\mathrm{rib}}$ is set to be 0.566, which is the critical density obtained from the tensile strength model for direct compression [6]. Similarly, the upper bound for the mean granule size is set according to the ASTM D4767 standard, which indicates that for proper die filling and consistent tablet properties, the mean particle size must be less than one-sixth of the die diameter *D*. The lower bound for the mean granule size is set to zero because the crystal size of APAP (35 μm) and MCC PH102 (100 μm) are small compared to mean granule sizes. Table 3 summarizes the first step of the proposed approach.

Model parameters ξ of the tableting ROMs M(ξ), except for tablet weight, are then modeled as bivariate rational functions of *X* and *Y* (see Table 4). In turn, the tableting models presented in Section 3 depend on the dry granulation CPP|CMA, as follows

(25) $$F_{\mathrm{punch}}=\frac{\pi D^{2}\left(\rho^{\mathrm{in}-\mathrm{die}}-\rho_{c}\left(X,Y\right)\right)}{4b\left[\rho^{\mathrm{in}-\mathrm{die}}\left(a\left(X,Y\right)-1\right)+\rho_{c}\left(X,Y\right)\right]}$$

(26) $$\epsilon_{\rho }=\epsilon_{0}\left(X,Y\right){\left(\frac{\rho^{\mathrm{in}-\mathrm{die}}-\rho_{c,\epsilon }\left(X,Y\right)}{1-\rho_{c,\epsilon }\left(X,Y\right)}\right)}^{n}$$

(27) $$\sigma_{t}=\sigma_{0}\left(X,Y\right)\left[1-\left(\frac{1-\rho^{\mathrm{tablet}}}{1-\rho_{c,\sigma_{t}}\left(X,Y\right)}\right)e^{\left(\rho^{\mathrm{tablet}}-\rho_{c,\sigma_{t}}\left(X,Y\right)\right)}\right].$$

Lastly, a model library F is built by progressively simplifying the bivariate rational function f(X,Y) in Equation (16). Specifically, nine rational functions, presented in Table 5, are considered. For simplicity and computational efficiency, the model library F contains only twenty-five pairs (one for each model parameter in ξ; see Table 4), out of all eighty-one possible combinations. The models in F are presented in Table 6.

The integration of tableting ROMs for die filling and tablet weight with dry granulation can be pursued, borrowing mechanistic insight from studies of the packing fraction of polydisperse powders. Specifically, for the same turret and feeder speeds, we propose that the filling efficacy η in Equation (9) only depends on changes in the polydispersity of the bimodal size distribution of the granules produced by the roller compactor (see Section 2.1), i.e.,

(28) $$W=\eta \rho_{b}\;V_{\mathrm{fill}}\equiv \phi \left(X,Y\right)\rho_{t}\rho^{\mathrm{ribbon}}\;V_{\mathrm{fill}},$$

where ϕ(X,Y) is the packing fraction and the normalized variables *X* and *Y* depend on the mean size of small and large modes of the bimodal Weibull distribution, i.e., on $\mu_{1}$ and $\mu_{2}$, respectively, and the fraction of the small mode α. Brouwers and co-workers proposed an analytical expression for the packing fraction of bimodal hard monodispersed spheres arranged in crystalline structures [29] and randomly packed [30]. For $\mu_{2}/\mu_{1}$ being small and equal to the size ratio of the two modes, the proposed relation is given by

(29) $$\phi \left(\mu_{2}/\mu_{1},\alpha \right)=\frac{\phi^{\mathrm{rp}}\left\{\left(1-\alpha \right)\left[1-{\left(\mu_{2}/\mu_{1}\right)}^{3}\right]+{\left(\mu_{2}/\mu_{1}\right)}^{3}\right\}}{C\;\left(1-\alpha \right)\alpha \left[{\left(\mu_{2}/\mu_{1}\right)}^{3}-1\right]+\left(1-\alpha \right)\left[(1-{\left(\mu_{2}/\mu_{1}\right)}^{3})\right]+{\left(\mu_{2}/\mu_{1}\right)}^{3}},$$

where $\phi^{\mathrm{rp}}$ is the random packing fraction of unimodal spheres, and *C* is a constant that depends on the mode of random packing (e.g., loose, close). We extend this relationship to a bimodal Weibull distribution of non-spherical deformable granules by retaining the mathematical form and relaxing constraints on model parameters, that is,

(30) $$\phi \left(X,Y\right)=\frac{p_{2}X+p_{4}}{q_{1}XY+q_{2}X+1},$$

with $X=\left(1-\alpha \right)\left(1-\mu_{1}^{3}/\mu_{2}^{3}\right)\in \left[0,1\right]$ and Y=α∈[0,1]—which have the same order of magnitude, i.e., they are normalized. It is worth noting that for α=1 and α=0, the mixture becomes unimodal. If the modes are monodispered [30], then the packing fraction is $\phi^{\mathrm{rp}}$. If the modes correspond to Weibull distributions, we propose that the packing fraction is $p_{4}$, that is, we constrain $p_{2}$ to be equal to $q_{2}p_{4}$. Therefore, for tablet weight, the model library F has only one model with $p_{1}=p_{3}=q_{3}=0$, $r_{X}=r_{Y}=1$, and $p_{2}=q_{2}p_{4}$. Meng et al. [31] show an increase in packing fraction with a decrease in the particle size ratio $\mu_{1}/\mu_{2}$, for any value of α and sizes of the same order of magnitude (here assumed valid for $\mu_{1}/\mu_{2}\in \left[0.2,1\right]$), that is, $\phi \geq \phi^{\mathrm{rp}}$, as reported for other mixtures [30,32].

## 6. Results and Discussion

The CMAs, CPPs, and CQAs measured and collected as part of the experimental campaign described in Section 2 were used to identify ROMs and their parameters for integrating dry granulation and tableting processes. Specifically, the models presented in Section 5 were adopted, i.e., the models that emerged from the systematic application of the model selection and parameter estimation approach proposed in Section 4 to the tableting ROMs presented in Section 3. The weight, dimensions, and tensile strength were measured for a total number of 2400 tablets, corresponding to 24 different tableting conditions, under which the compaction force, dosing position, and in-die main compression thickness are available.

The resulting ROMs for each of the four stages of compaction are presented next, in turn.

### 6.1. Tablet Weight

**Figure 6:**
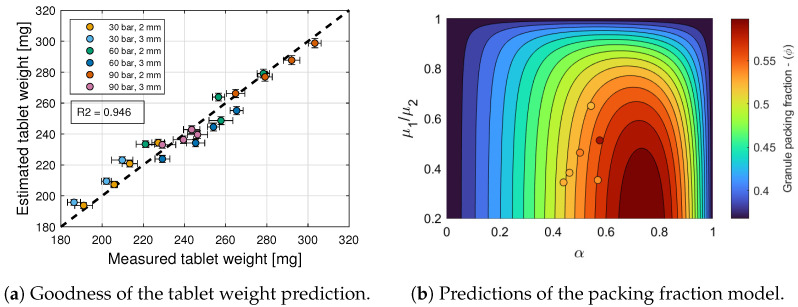
Tablet weight model.

The model library F has only one model, given by

(31) $$\phi \left(X,Y\right)=p_{4}\frac{q_{2}X+1}{q_{1}XY+q_{2}X+1},$$

with $X=\left(1-\alpha \right)\left(1-\mu_{1}^{3}/\mu_{2}^{3}\right)$ and Y=α. The inequality constraints D are given by $p_{4}>0$ and $q_{1}<0$, since $\phi \left(X,Y\right)\geq p_{4}>0$. Figure 6a shows that the goodness of the tablet weight prediction is high, with $R^{2}=0.946$. Figure 6b shows predictions of this packing fraction model for tablets formed with granules of different size ratios $\mu_{1}/\mu_{2}$ and fractions of the small mode α. It is evident from this figure that the packing density is maximum when the fraction α of the small mode is around 0.7. Furthermore, for values of α between 0.6 and 0.9, the packing density increases as the size ratio $\mu_{1}/\mu_{2}$ decreases. This region of the design space is highly sensitive to changes in the operating conditions of the roller compactor.

The measured tablet weight exhibits a relative standard deviation equal to 1.6% on an average across the different 24 tableting conditions. Hence, the weight is modeled by a normal distribution with a mean equal to (28), using (31), and with a relative standard deviation equal to 1.6%. In turn, the standard deviation of the CQAs and CPPs for the other three stages of compaction will be estimated using a simple Monte Carlo approach and 10,000 weight samples. Specifically, compaction force, tablet density, and tensile strength are estimated as normal distributions with mean and standard deviations resulting from the Monte Carlo analysis.

### 6.2. Compaction Force

**Figure 7:**
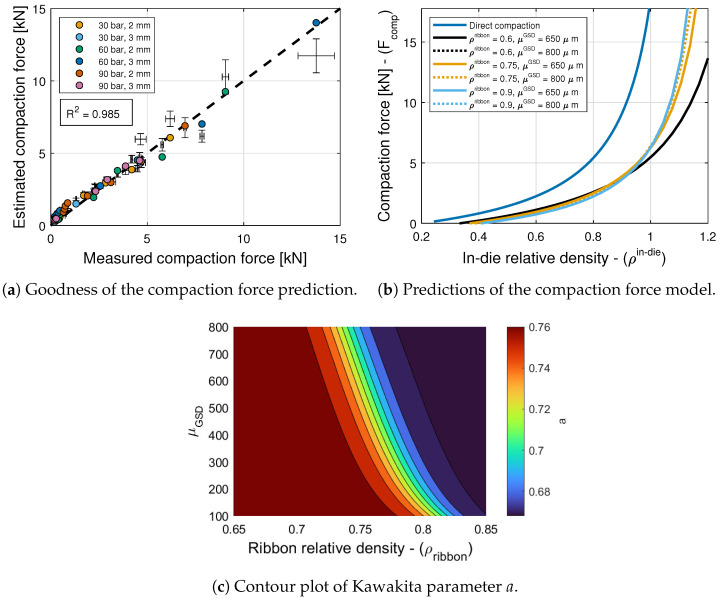
Compaction force model.

The model selection and parameter estimation approach proposed in Section 4 identifies model (7,7) as optimal within the model library F for *a* and $\rho_{c}$ (see Table 6, Table 7 and Table 11), with $X={\left[\left(\rho^{\mathrm{ribbon}}-\rho_{c,\mathrm{rib}}\right)/\left(1-\rho^{\mathrm{ribbon}}\right)\right]}^{r_{X}}$ and $Y={\left[\mu^{\mathrm{GSD}}/\left(D/6-\mu^{\mathrm{GSD}}\right)\right]}^{r_{Y}}$. The inequality constraints D are given by $p_{i}>0,q_{j}>0$ with i=1,4,j=1, and r∈(0,10]. The total number of model parameters is then $N_{p}=9$, i.e., 3 parameters for *a*; 3 parameters for $\rho_{c}$; $r_{X}$ and $r_{Y}$; and parameter *b*. Furthermore, the nonlinear constraints $\rho^{\mathrm{in}-\mathrm{die}}-\rho_{c}>0$ and $\rho^{\mathrm{in}-\mathrm{die}}\left(a-1\right)+\rho_{c}>0$ are imposed to ensure that the estimated compaction force is positive, for the values of $\rho^{\mathrm{in}-\mathrm{die}}$ used experimentally. Figure 7a shows with symbols that the goodness of the compaction force prediction is high, with $R^{2}=0.985$. Figure 7b shows predictions of this model for tablets formed with granules of different ribbon densities and different mean granule sizes. The figure also shows predictions of the model proposed by Bachawala and Gonzalez [6] for tablets formed by direct compaction using 10% APAP, 90% MCC, and no lubricant or glidant. It is evident from the figure that the compaction force is lower for granules with a lower ribbon density, and significantly lower compared to the tablets formed using direct compaction.

The critical ribbon density, i.e., the smallest density at which a ribbon is formed by the roller compactor, is fixed to be a constant $\rho_{c,\mathrm{rib}}=0.566$, which is the smallest tablet density at which a tablet with adequate strength is formed by direct compaction (i.e., $\rho_{c,\sigma_{t}}=0.566$ from Bachawala and Gonzalez [6]). This value is also used in the elastic recovery and tensile strength models.

The critical or jamming relative density $\rho_{c}$ varies slightly for different granules and it is greater than the critical density observed in direct compaction, which can be attributed to a granule size distribution that packs and consolidates better than the wide GSD of 10% APAP and 90% MCC used in direct compaction. In contrast, the Kawakita parameter *a*, which is known to approximate the total degree of compression or total compressibility of the granular system [23,33], varies significantly for different granules (see Figure 7c). Specifically, it is evident from the figure that there is a strong dependency on ribbon density $\rho^{\mathrm{ribbon}}$ and a weak dependency on the mean granule size $\mu^{\mathrm{GSD}}$. The value of *a* decreases as the granule density increases, i.e., the ability of the granules to compress decreases as more plastic work is imparted during granulation [34,35]. Additionally, the product of Kawakita parameters *a* and *b* is known to approximate the degree of particle rearrangement during compression. The degree of particle rearrangement for direct compression (a=0.824 and b=97.38 ${\mathrm{GPa}}^{-1}$) is higher compared to the granules studied here (a∈[0.66,0.76] and b=61.39 ${\mathrm{GPa}}^{-1}$ from Figure 7c), which can be reconciled with the observation that the critical or jamming density for direct compression is smaller than the one for the granulated material.

Next, the tablet weight model, i.e., Equations (28) and (31), and the compaction force model, i.e., Equation (25), are composed using the Monte Carlo approach to generate 10,000 force predictions for each tableting condition. The mean and standard deviation values of the estimates are shown in Figure 7a along with the standard deviation in the measurements. It is interesting to note that the estimated and measured standard deviations are similar for most tableting conditions. This suggests that the range of measured compaction forces can be attributed to variability in tablet weight. Naturally, the figure also shows that errors in the composed models are compounded cf. the symbols estimated from the measured weight with standard deviation bounds obtained from an estimated weight distribution.

### 6.3. Elastic Recovery and Tablet Density

**Figure 8:**
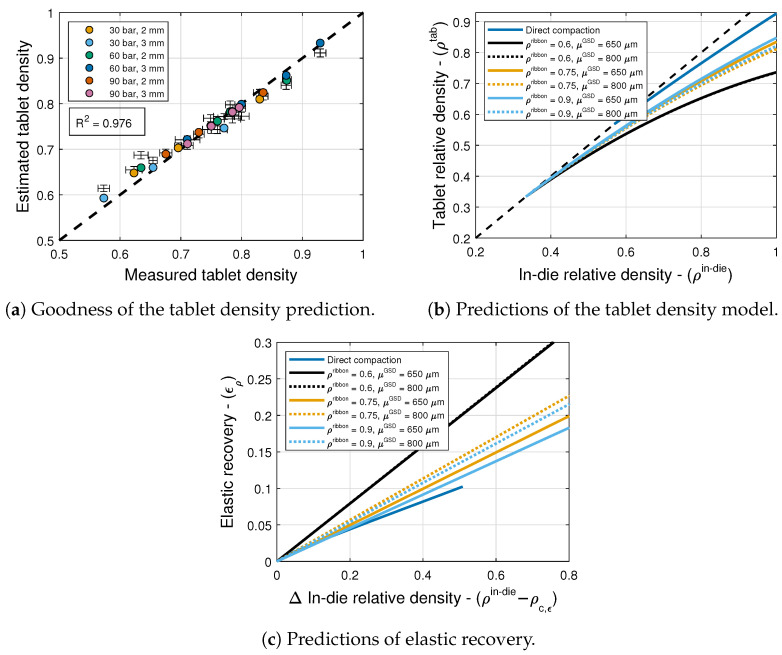
Tablet density and elastic recovery models.

The model selection and parameter estimation approach proposed in Section 4 identifies model (4,9) as optimal within the model library F for $\epsilon_{0}$ and $\rho_{c,\epsilon }$ (see Table 6, Table 8 and Table 11), with $X={\left[\left(\rho^{\mathrm{ribbon}}-\rho_{c,\mathrm{rib}}\right)/\left(1-\rho^{\mathrm{ribbon}}\right)\right]}^{r_{X}}$ and $Y={\left[\mu^{\mathrm{GSD}}/\left(D/6-\mu^{\mathrm{GSD}}\right)\right]}^{r_{Y}}$. The inequality constraints D are given by $p_{i}>0,q_{j}>0$ with i=1,2,4,j=1,2, and r∈(0,10]. Furthermore, the constraints $0<p_{i}<q_{i}$ with i=1,2,3,4 are imposed to ensure $\epsilon_{0}\in \left(0,1\right)$, and $\rho_{c,\epsilon }\in \left({\bar{\rho }}_{c},1\right)$, with ${\bar{\rho }}_{c}$ being the lower bound of the compaction critical relative density, to ensure consistency. Figure 8a shows with symbols that the goodness of the tablet density prediction is high, with $R^{2}=0.976$. Figure 8b shows predictions of this model for tablets formed with granules of different ribbon densities and mean granule sizes. The figure also shows predictions of the model proposed by Bachawala and Gonzalez [6] for tablets formed by direct compaction using 10% APAP, 90% MCC, and no lubricant or glidant.

The onset of elastic recovery $\rho_{c,\epsilon }=0.334$ is constant for all granules. This study suggests that elastic recovery, i.e., $\epsilon_{\rho }=1-\rho^{\mathrm{tablet}}/\rho^{\mathrm{in}-\mathrm{die}}$, is sensitive to both ribbon density and mean granule size. Specifically, lower elastic recovery is observed in tablets made from granules of a smaller size (cf. solid and dashed lines in Figure 8c) and with higher density (cf. different colors in Figure 8c). Additionally, compared to direct compaction, the elastic recovery of tablets formed through dry granulation is significantly higher, resulting in tablets with lower relative density. This can be attributed to the brittle nature of the granules [34].

Next, the tablet weight model, i.e., Equations (28) and (31), and the elastic recovery model, i.e., Equation (26), are composed using the Monte Carlo approach to generate 10,000 tablet density predictions for each tableting condition. The mean and standard deviation values of the estimates are shown in Figure 8a along with the standard deviation in the measurements. It is interesting to note that the estimated and measured standard deviations are similar for most tableting conditions. As was the case for the compaction force, this suggests that the range of measured tablet densities can be attributed to variability in tablet weight. In this case, however, the figure indicates that compounding errors do not have a strong impact on the mean values, cf. the symbols estimated from the measured tablet density with standard deviation bounds obtained from an estimated tablet density distribution.

### 6.4. Tensile Strength

The model selection and parameter estimation approach proposed in Section 4 identifies model (1,1) as optimal within the model library F for $\sigma_{0}$ and $\rho_{c,\sigma }$ (see Table 6, Table 9 and Table 11), with $X={\left[\left(\rho^{\mathrm{ribbon}}-\rho_{c,\mathrm{rib}}\right)/\left(1-\rho^{\mathrm{ribbon}}\right)\right]}^{r_{X}}$ and $Y={\left[\mu^{\mathrm{GSD}}/\left(D/6-\mu^{\mathrm{GSD}}\right)\right]}^{r_{Y}}$. The inequality constraints D are $p_{i}>0,q_{j}>0$ with i=1,2,3,4,j=1,2,3, and r∈(0,10]. Furthermore, the nonlinear constraints $\rho_{c,\sigma }>\rho_{c,\epsilon }$ are imposed to ensure that the tensile strength values are positive for the values of $\rho^{\mathrm{tablet}}$ used experimentally. However, the goodness of this model is poorer than that of previous models, cf. $R^{2}=0.906$ with other values. This observation calls for the examination of the results from Nordström et al. [36] showing that tensile strength depends on the size ratio and the percentage of large particles, rather than the mean granule size, and that these two effects are coupled with each other. In contrast, studies on the effect of granule size on the tensile strength of tablets are mostly limited to monodisperse granules or narrow sieve cuts with different mean granule sizes. For example, Herting and Kleinebudde [9] showed that smaller sieve sizes of MCC granules lubricated with magnesium stearate form stronger tablets, and Mitra et al. [34] observed that the granule size has no significant effect on the tensile strength of granules.

**Figure 9:**
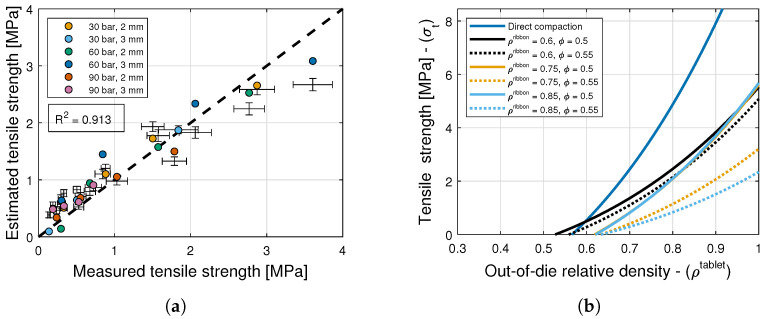
Tensile strength model. (**a**) Goodness of the tablet tensile strength prediction. (**b**) Predictions of the tablet tensile strength model.

Therefore, following the approach presented in Section 4, the model library is expanded with models that depend on $X={\left[\left(\rho^{\mathrm{ribbon}}-\rho_{c,\mathrm{rib}}\right)/\left(1-\rho^{\mathrm{ribbon}}\right)\right]}^{r_{X}}$ and $Z={\left[\left(\phi -\phi_{\min}\right)/\left(\phi_{\max}-\phi \right)\right]}^{r_{Z}}$, i.e., on ribbon density and packing fraction ϕ. In turn, the (1,1) model added to the library emerges as optimal with $R^{2}=0.9103$ (see Table 9 and Table 11 and Figure 9a). Figure 9b shows predictions of this model for tablets formed with granules of different ribbon densities and mean granule sizes. It is evident from this figure that a higher initial packing fraction results in a lower tablet strength, which is in agreement with observations from Johansson and Alderborn [37] indicating that higher initial bulk densities of MCC granules lead to tablets with lower tensile strength. Similarly, a higher granule relative density results in lower tensile strength despite the low elastic recovery. This is in agreement with observations from Nordström and Alderborn [38] for MCC granules. The figure also shows predictions of the model proposed by Bachawala and Gonzalez [6] for tablets formed by direct compaction using 10% APAP, 90% MCC, and no lubricant or glidant. Evidently, there is a loss in tabletability as a result of the dry granulation process. The powder is subjected to plastic work while the ribbon is formed inside the roller compactor, and it thus loses its ability to be further compacted during tableting to form inter-granule interfaces with enough bonding strength [39,40].

Next, the tablet weight model, i.e., Equations (28) and (31), the elastic recovery model, i.e., Equation (26), and the tensile strength model, i.e., Equation (27), are composed using the Monte Carlo approach to generate 10,000 tensile strength predictions for each tableting condition. The mean and standard deviation values of the estimates are shown in Figure 9a along with the standard deviation in the measurements. It is interesting to note that the estimated standard deviations are smaller than the measured values for most tableting conditions. In contrast to the compaction force and tablet density, this suggests that the range of measured tensile strength values cannot solely be attributed to variability in tablet weight.

### 6.5. Discussion

The ROMs presented above are based on product and process understanding, and their integration with dry granulation offers the opportunity to gain insight into the granule properties that have a dominant effect on each of the four stages of compaction. Hence, for each CPP and CQA, we examine which of the granule properties (namely the ribbon density $\rho^{\mathrm{ribbon}}$ and the granule size distribution characterized by the mean granule size $\mu^{\mathrm{GSD}}$, the size ratio $\mu_{1}/\mu_{2}$, or the fraction of the small mode α) appear consistently in the top five ranked models. This granule property is said to have the most dominant effect or first-order effect on the observed CPP or CQA. The first- and second-order effects of granule properties on tableting CPPs and CQAs are listed in Table 10. The tablet weight is more sensitive to the size ratio $\mu_{1}/\mu_{2}$ and linearly proportional to ribbon density. On the other hand, the compaction force, elastic recovery, and tensile strength are more sensitive to the ribbon density compared to the average granule size. Yohannes et al. [41] showed that the elastic recovery, compaction force, and tensile strength were not affected by the amount of fines in the powder bed. Perez-Gandarillas et al. [42] reported that the tablet properties were significantly affected by roll compaction, but not the milling stage. Therefore, ribbon density prevails over granule size when it comes to the compaction force, elastic recovery, and tensile strength of tablets, which is in agreement with model predictions. In fact, for all six granules, the mean size of the small mode varies more than the mean size of the large mode (cf. ±15% and ±6%, and see Table 1). Interestingly, the second-order effect of granule size on the tensile strength is through the packing fraction, and thus is mainly through the size ratio.

**Table 10:** First- and second-order effects of granule properties on tableting CPPs and CQAs.

Granule Properties	Tablet Weight	Compaction Force	Elastic Recovery	Tensile Strength
$\rho^{\mathrm{ribbon}}$	1st	1st	1st	1st
$\mu^{\mathrm{GSD}}$	—	2nd	2nd	—
$\mu_{1}/\mu_{2}$	1st	—	—	2nd
α	2nd	—	—	—

**Table 11:** Summary of the best models for tablet CQAs and CPPs with the corresponding parameters.

**Weight**	$W=\phi \rho_{t}\rho^{\mathrm{ribbon}}\;V_{\mathrm{fill}}$
$X=\frac{1-\alpha }{1-\mu_{1}^{3}/\mu_{2}^{3}}$ , Y=α	
$\phi =p_{4}\frac{q_{2}X+1}{q_{1}XY+q_{2}X+1}$	$p_{4}=0.367$; $q_{1}=6.08$; $q_{2}=7.33$
$X={\left(\frac{\rho^{\mathrm{ribbon}}-\rho_{c,\mathrm{rib}}}{1-\rho^{\mathrm{ribbon}}}\right)}^{r_{X}}$ , $Y={\left(\frac{\mu^{\mathrm{GSD}}}{D/6-\mu^{\mathrm{GSD}}}\right)}^{r_{Y}}$	$\rho_{c,\mathrm{rib}}=0.566$
$Z={\left(\frac{\phi -\phi_{\min}}{\phi_{\max}-\phi }\right)}^{r_{Z}}$	$\phi_{\min}=0.367$; $\phi_{\max}=0.631$
**Compaction force**	$F_{\mathrm{punch}}=\frac{\pi D^{2}\left(\rho^{\mathrm{in}-\mathrm{die}}-\rho_{c}\right)}{4b\left(\rho^{\mathrm{in}-\mathrm{die}}\left(a-1\right)+\rho_{c}\right)}$
	$r_{X}=10$; $r_{Y}=2.39$
	$b=61.39\;{\mathrm{GPa}}^{-1}$
$a=\frac{p_{1}XY+p_{4}}{q_{1}XY+1}$	$p_{1}=18.50$ ; $q_{1}=27.69$ $p_{4}=0.765$
$\rho_{c}=\frac{p_{1}XY+p_{4}}{q_{1}XY+1}$	$p_{1}=8.73$ ; $q_{1}=21.38$ $p_{4}=0.33$
**Elastic recovery**	$\epsilon_{\rho }=\epsilon_{0}{\left(\frac{\rho^{\mathrm{in}-\mathrm{die}}-\rho_{c,\epsilon }}{1-\rho_{c,\epsilon }}\right)}^{n}$
	$r_{X}=1$; $r_{Y}=1$
	n=0.472
$\epsilon_{0}=\frac{p_{1}XY+p_{2}X+p_{4}}{q_{1}XY+q_{2}X+1}$	$p_{1}=5.04$; $q_{1}=18.78$ $p_{2}=0.243$; $q_{2}=16.10$ $p_{4}=0.597$
$\rho_{c,\epsilon }=p_{4}$	$p_{4}=0.334$
**Tensile strength**	$\sigma_{t}=\sigma_{0}\left[1-\left(\frac{1-\rho^{\mathrm{tablet}}}{1-\rho_{c,\sigma_{t}}}\right)e^{\left(\rho^{\mathrm{tablet}}-\rho_{c,\sigma_{t}}\right)}\right]$
	$r_{X}=8.26$; $r_{Z}=1.98$
$\sigma_{0}=\frac{p_{1}XZ+p_{2}X+p_{3}Z+p_{4}}{q_{1}XZ+q_{2}X+q_{3}Z+1}$	$p_{1}=0.33\;\mathrm{MPa}$ ; $q_{1}=1.79$ $p_{2}=124.23\;\mathrm{MPa}$ ; $q_{2}=8.90$ $p_{3}=0.18\;\mathrm{MPa}$ ; $q_{3}=0.04$ $p_{4}=5.90\;\mathrm{MPa}$
$\rho_{c,\sigma_{t}}=\frac{p_{1}XZ+p_{2}X+p_{3}Z+p_{4}}{q_{1}XZ+q_{2}X+q_{3}Z+1}$	$p_{1}=50.92\;\mathrm{MPa}$ ; $q_{1}=79.66$ $p_{2}=14.82\;\mathrm{MPa}$ ; $q_{2}=41.28$ $p_{3}=0.02\;\mathrm{MPa}$ ; $q_{3}=0.02$ $p_{4}=0.51\;\mathrm{MPa}$

## 7. Conclusions

We have presented a systematic and automatic approach for integrating tableting reduced-order models with upstream unit operations. The approach not only identifies the upstream critical material attributes and process parameters that describe the coupling to the first and second orders, but it also selects the mathematical form of such coupling and estimates its parameters. Specifically, we have proposed that the coupling can be generally described by normalized bivariate rational functions. By restricting attention to a library comprising a finite number of functions, we have posed model selection and parameter identification as a trade-off between model complexity (i.e., the number of model parameters) and the goodness of the model prediction (i.e., the sum of squared errors) using the Akaike Information Criterion. We have demonstrated this approach for dry granulation, a unit operation commonly used to enhance the flowability of pharmaceutical powders by improving the granule size distribution, which, inevitably, negatively impacts the tabletability by reducing particle porosity and imparting plastic work. Granules of different densities and size distributions were made with a 10% *w*/*w* acetaminophen and 90% *w*/*w* microcrystalline cellulose formulation, and tablets with a wide ranges of relative densities were fabricated using an Alexanderwerk WP120 roller compactor and a Natoli NP-400 tablet press. Since our approach is based on product and process understanding, it is not only essential to enabling the end-to-end integration, control, and optimization of dry granulation and tableting processes, but it also offers insights into the granule properties that have a dominant effect on each of the four stages of powder compaction, namely die filling, compaction, unloading, and ejection. Specifically, for each CPP and CQA, we have examined which of the granule properties (namely the ribbon density $\rho^{\mathrm{ribbon}}$ and the granule size distribution characterized by the mean granule size $\mu^{\mathrm{GSD}}$, the size ratio $\mu_{1}/\mu_{2}$, or the fraction of the small mode α) appear consistently in the top five ranked models in the library, i.e., we have identified the most dominant effect or first-order effect on the observed CPP or CQA. The tablet weight was observed to be more sensitive to the size ratio and linearly proportional to the ribbon density. On the other hand, the compaction force, elastic recovery, and tensile strength were observed to be more sensitive to the ribbon density compared to the average granule size. Interestingly, the second-order effect of the tensile strength on the granule size was rather observed to be through the packing fraction, and thus mainly through the size ratio. It is worth noting that these CMAs are available at-line from indirect measurements using NIR monitoring and high-speed imaging and/or from soft sensors built from hybrid mechanistic/ML-based models that use CPP measurements. Therefore, by building redundancy in the PAT sensor network, model-based data reconciliation based on process understanding emerges as an effective real-time process management tool for accomplishing robust process monitoring and control.

## 8. Future Work

The limitations of the proposed methodology and the corresponding mitigation strategies for future work are discussed below.

Firstly, the direct compaction tableting ROMs presented in Section 3 are optimal for formulations with elasto-plastic properties similar to MCC. For materials with different compaction profiles, such as lactose [43,44], these ROMs must be replaced with models that more accurately capture the relevant compaction trends [45,46,47]. Secondly, we identified two upstream CMAs, namely ribbon density and granule size, that impact the tablet CQAs. However, other relevant CMAs must be considered for different formulations or upstream unit operations, such as formulations with a lubricant that require mixing and blending [6]. Furthermore, if more than two CMAs were identified as required to describe the first- and second-order coupling, the bivariate rational function would then need to be replaced with a multivariate rational function. Lastly, we considered $\mu^{\mathrm{GSD}}$ and $\mu_{1}/\mu_{2}$ as the representative parameters of the granule size distribution. While this is appropriate for a bimodal distribution, other particle/granule size or shape characteristics should be identified for more complex formulations and processes.

We close by pointing out that, given the nonlinear nature of these models, real-time control in continuous solids processing is typically implemented using moving-horizon estimation and nonlinear model predictive control (MHE-NMPC) rather than traditional PID control. For example, Huang et al. [4,8] demonstrated MHE-NMPC control of the direct compaction route when a glidant is added to the blend (see Figure 1 for an illustration of this process). Specifically, the ROMs developed by Bachawala and Gonzalez [6] were used to predict the tablet weight, compaction force, and production rate. Huang et al. [8] integrated these predictions into the NMPC framework to control the tablet press’ dosing position, compression thickness, and turret speed. Similarly, predictions made by the ROMs described in this work can be used to control the tablet press using an NMPC framework. In addition, Huang et al. [11] implemented an NMPC framework for the roller compactor using a hybrid model for ribbon density and granule size. Therefore, one can speculate that end-to-end continuous control can be demonstrated using an MHE-NMPC framework that combines the tableting ROMs proposed here and the roller compactor hybrid model. Alternatively, future efforts may also consider incorporating in-line measurements of ribbon density and granule size using sensors such as Innopharma Multieye2 and SentroPAT, respectively.

## Figures and Tables

**Figure 5:**
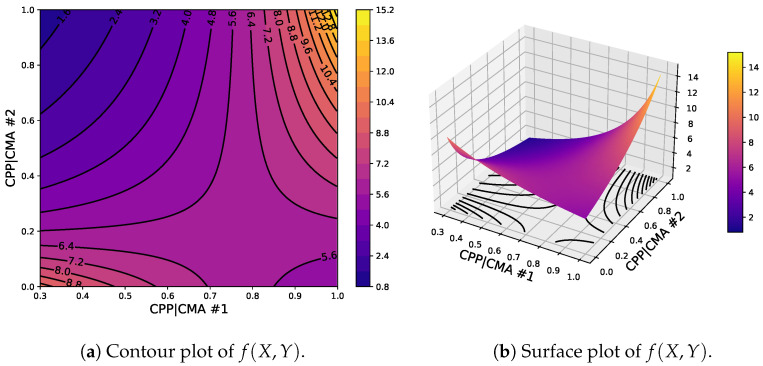
Contour and surface plots of ξ(CPP|CMA;θ) with {CPP|CMA#1,CPP|CMA#2}≡{x,y}∈[0.3,1]×[0,1]} and $\theta =\left\{p1,p2,p3,p4,q1,q2,q3,r_{X},r_{Y}\right\}=\left\{15,10,5,10,1,2,4,1,1\right\}$.

**Table 1:** Hybrid model parameters for the six different granules, and the corresponding granule size of the small mode, the large mode, and the mean granule size.

Roll Pressure [bar]	Roll Gap [mm]	α	$\lambda_{1}$ [μm]	$\lambda_{2}$ [μm]	$k_{1}$	$k_{2}$	$\mu_{1}$ [μm]	$\mu_{2}$ [μm]	$\mu^{\mathrm{GSD}}$ [μm]	$\rho^{\mathrm{ribbon}}$
30	2	0.54	524	1055	2.25	5.32	457	991	696	0.640
30	3	0.57	518	1019	2.25	5.43	325	999	667	0.613
60	2	0.46	541	1170	2.24	4.98	479	1074	800	0.739
60	3	0.58	517	1009	2.26	5.46	458	931	658	0.708
90	2	0.44	546	1202	2.23	4.89	484	1102	830	0.803
90	3	0.50	533	1114	2.24	5.15	472	1024	747	0.770

**Table 2:** Roller compactor and tablet press setpoints for different granules and tablets.

	Roller Compactor Settings		Tablet Press Settings	
**Batch**	**Roll Pressure [bar]**	**Roll Gap [mm]**	**Dosing Position [mm]**	**Main Compression Thickness [mm]**
1	30	2	7.0–8.5	2.5–3.0
2	30	3	7.0–8.0	2.5–3.2
3	60	2	7.5–9.0	2.5–3.0
4	60	3	7.0–8.0	2.5–3.2
5	90	2	8.0–9.0	3.5–4.0
6	90	3	7.0–7.3	3.0–3.3

**Table 3:** Normalization of the mean granule size $\mu^{\mathrm{GSD}}$ and ribbon relative density $\rho^{\mathrm{ribbon}}$.

CPP|CMA	Lower Bound (lb)	Upper Bound (ub)	Normalized CPP|CMA
Ribbon relative density: $\rho^{\mathrm{ribbon}}$	$\rho_{c,\mathrm{rib}}$	1	$X={\left(\frac{\rho^{\mathrm{ribbon}}-\rho_{c,\mathrm{rib}}}{1-\rho^{\mathrm{ribbon}}}\right)}^{r_{X}}$
Mean granule size: $\mu^{\mathrm{GSD}}$	0	D/6	$Y={\left(\frac{\mu^{\mathrm{GSD}}}{D/6-\mu^{\mathrm{GSD}}}\right)}^{r_{Y}}$

**Table 4:** ROM parameters, ξ, modeled as bivariate rational functions of *X* and *Y*.

	Compaction Force	Elastic Recovery	Tensile Strength
M	$F_{\mathrm{punch}}$	$\epsilon_{\rho }$	$\sigma_{t}$
ξ	a(X,Y)	$\epsilon_{0}\left(X,Y\right)$	$\sigma_{0}\left(X,Y\right)$
	$\rho_{c}\left(X,Y\right)$	$\rho_{c,\epsilon }\left(X,Y\right)$	$\rho_{c,\sigma_{t}}\left(X,Y\right)$

**Table 5:** Constrained variants of the normalized bivariate rational function f(X,Y).

Model Number	Rational Function	No. of Parameters
1	f(X,Y)	9
2	$r_{X}=r_{Y}=1$	7
3	$p_{3}=q_{3}=0$	7
4	$p_{3}=q_{3}=0,r_{X}=r_{Y}=1$	5
5	$p_{2}=q_{2}=0$	7
6	$p_{2}=q_{2}=0,r_{X}=r_{Y}=1$	5
7	$p_{2}=p_{3}=q_{2}=q_{3}=0$	5
8	$p_{2}=p_{3}=q_{2}=q_{3}=0,r_{X}=r_{Y}=1$	3
9	Constant − $f\left(X,Y\right)=p_{4},r_{X}=r_{Y}=1$	1

**Table 6:** Model library F: pairs of constrained variants of bivariate rational functions used by compaction force, elastic recovery, and tensile strength.

Function Variant	1	2	3	4	5	6	7	8	9
1	✓								✓
2		✓							✓
3			✓						✓
4				✓					✓
5					✓				✓
6						✓			✓
7							✓		✓
8								✓	✓
9	✓	✓	✓	✓	✓	✓	✓	✓	✓

**Table 7:** Top five best models within the model library F for parameters *a* and $\rho_{c}$ of the compaction force ROM (see Table 5 and Table 6).

a(X,Y)	$\rho_{c}\left(X,Y\right)$	$N_{p}$	SSE	$R^{2}$	AIC
7	7	9	5.539	0.985	−79.37
6	9	7	6.707	0.982	−74.57
4	9	7	6.79	0.982	−74.00
4	4	11	5.903	0.984	−72.44
8	8	7	7.053	0.981	−72.26

**Table 8:** Top five best models within the model library F for parameters $\epsilon_{0}$ and $\rho_{c,\epsilon }$ of the tablet density and elastic recovery ROM (see Table 5 and Table 6).

$\epsilon_{0}\left(X,Y\right)$	$\rho_{c,\epsilon }\left(X,Y\right)$	$N_{p}$	SSE	$R^{2}$	AIC
4	9	7	0.004	0.976	−185.5
8	9	5	0.005	0.968	−182.6
2	9	9	0.004	0.977	−181.6
3	9	9	0.004	0.976	−181.5
6	9	7	0.005	0.970	−180.3

**Table 9:** Top five best models within the model library F for parameters $\sigma_{0}$ and $\rho_{c,\sigma }$ of the tensile strength ROM (see Table 5 and Table 6).

$\sigma_{0}\left(X,Y\right)$	$\rho_{c,\sigma_{t}}\left(X,Y\right)$	$\sigma_{0}\left(X,Z\right)$	$\rho_{c,\sigma_{t}}\left(X,Z\right)$	$N_{p}$	SSE	$R^{2}$	AIC
		1	1	0.913	177	16	−5302
1	1			0.906	193	16	−5123
		3	3	0.903	197	12	−5082
3	3			0.900	205	12	−5004
		7	7	0.881	243	8	−4639
		2	2	0.873	259	14	−4497
		5	5	0.873	260	12	−4491
1	9			0.862	281	10	−4327
2	2			0.856	293	14	−4229
4	4			0.848	309	10	−4122

## Data Availability

The original contributions presented in this study are included in the article; any further inquiries can be directed to the corresponding authors.

## Abbreviations

The following abbreviations are used in this manuscript:

QbD	Quality by Design
FDA	Food and Drug Administration
QbC	Quality by Control
API	Active Pharmaceutical Ingredients
CPP	Critical Process Parameters
CQA	Critical Quality Attributes
ROMs	Reduced-Order Models
NMPC	Nonlinear Model Predictive Control
CMAs	Critical Material Attributes
MCC	Microcrystalline Cellulose Avicel PH-102
APAP	Acetaminophen Grade 0048
GSD	Granule Size Distributions
ML	Machine-learning
NIR	Near-infrared

## References

[1] Su Q., Bommireddy Y., Shah Y., Ganesh S., Moreno M., Liu J., Gonzalez M., Yazdanpanah N., O’Connor T., Reklaitis G.V. (2019). Data reconciliation in the Quality-by-Design (QbD) implementation of pharmaceutical continuous tablet manufacturing. Int. J. Pharm..

[2] Yu L.X., Amidon G., Khan M.A., Hoag S.W., Polli J., Raju G., Woodcock J. (2014). Understanding pharmaceutical quality by design. AAPS J..

[3] Su Q., Ganesh S., Moreno M., Bommireddy Y., Gonzalez M., Reklaitis G.V., Nagy Z.K. (2019). A perspective on Quality-by-Control (QbC) in pharmaceutical continuous manufacturing. Comput. Chem. Eng..

[4] Huang Y.S., Sheriff M.Z., Bachawala S., Gonzalez M., Nagy Z.K., Reklaitis G.V. (2021). Evaluation of a Combined MHE-NMPC Approach to Handle Plant-Model Mismatch in a Rotary Tablet Press. Processes.

[5] Gonzalez M. (2019). Generalized loading-unloading contact laws for elasto-plastic spheres with bonding strength. J. Mech. Phys. Solids.

[6] Bachawala S., Gonzalez M. (2022). Development of mechanistic reduced order models (ROMs) for glidant and lubricant effects in continuous manufacturing of pharmaceutical solid-dosage forms. 32nd European Symposium on Computer Aided Process Engineering.

[7] Ferdoush S., Gonzalez M. (2023). Semi-mechanistic reduced order model of pharmaceutical tablet dissolution for enabling Industry 4.0 manufacturing systems. Int. J. Pharm..

[8] Huang Y.S., Sheriff M.Z., Bachawala S., Gonzalez M., Nagy Z.K., Reklaitis G.V. (2022). Application of MHE-based NMPC on a Rotary Tablet Press under Plant-Model Mismatch. Computer Aided Chemical Engineering.

[9] Herting M.G., Kleinebudde P. (2008). Studies on the reduction of tensile strength of tablets after roll compaction/dry granulation. Eur. J. Pharm. Biopharm..

[10] Gavi E., Reynolds G.K. (2014). System model of a tablet manufacturing process. Comput. Chem. Eng..

[11] Huang Y.-S., Lagare R.B., Bailey P., Sixon D., Gonzalez M., Nagy Z.K., Reklaitis G.V. (2024). Hybrid model development and nonlinear model predictive control implementation for continuous dry granulation process. Comput. Chem. Eng..

[12] Razavi S.M., Gonzalez M., Cuitino A.M. (2015). General and mechanistic optimal relationships for tensile strength of doubly convex tablets under diametrical compression. Int. J. Pharm..

[13] Pishnamazi M., Casilagan S., Clancy C., Shirazian S., Iqbal J., Egan D., Edlin C., Croker D.M., Walker G.M., Collins M.N. (2019). Microcrystalline cellulose, lactose and lignin blends: Process mapping of dry granulation via roll compaction. Powder Technol..

[14] Matji A., Donato N., Gagol A., Morales E., Carvajal L., Serrano D.R., Worku Z.A., Healy A.M., Torrado J.J. (2019). Predicting the critical quality attributes of ibuprofen tablets via modelling of process parameters for roller compaction and tabletting. Int. J. Pharm..

[15] Huang Y.S., Sixon D., Bailey P., Lagare R.B., Gonzalez M., Nagy Z.K., Reklaitis G.V., Kokossis A.C., Georgiadis M.C., Pistikopoulos E. (2023). A Machine Learning-assisted Hybrid Model to Predict Ribbon Solid Fraction, Granule Size Distribution and Throughput in a Dry Granulation Process. 33rd European Symposium on Computer Aided Process Engineering.

[16] Johanson J.R. (1965). A Rolling Theory for Granular Solids. J. Appl. Mech..

[17] Gupta A., Peck G.E., Miller R.W., Morris K.R. (2005). Real-time near-infrared monitoring of content uniformity, moisture content, compact density, tensile strength, and young’s modulus of roller compacted powder blends. J. Pharm. Sci..

[18] Crowley M.E., Hegarty A., McAuliffe M.A., O’Mahony G.E., Kiernan L., Hayes K., Crean A.M. (2017). Near-infrared monitoring of roller compacted ribbon density: Investigating sources of variation contributing to noisy spectral data. Eur. J. Pharm. Sci..

[19] Ilari J.L., Martens H., Isaksson T. (1988). Determination of particle size in powders by scatter correction in diffuse near-infrared reflectance. Appl. Spectrosc..

[20] El Hagrasy A., Cruise P., Jones I., Litster J. (2013). In-line size monitoring of a twin screw granulation process using high-speed imaging. J. Pharm. Innov..

[21] Pitt K., Newton J., Stanley P. (1989). Stress distributions in doubly convex cylindrical discs under diametral loading. J. Phys. D Appl. Phys..

[22] Singh R., Román-Ospino A.D., Romañach R.J., Ierapetritou M., Ramachandran R. (2015). Real time monitoring of powder blend bulk density for coupled feed-forward/feed-back control of a continuous direct compaction tablet manufacturing process. Int. J. Pharm..

[23] Kawakita K., Lüdde K.H. (1971). Some considerations on powder compression equations. Powder Technol..

[24] Leuenberger H., Rohera B.D. (1986). Fundamentals of powder compression. I. The compactibility and compressibility of pharmaceutical powders. Pharm. Res..

[25] Cuyt A.A., Verdonk B.M. (1985). Multivariate rationale Interpolation. Computing.

[26] Lehmensiek R., Meyer P. (2000). An efficient adaptive frequency sampling algorithm for model-based parameter estimation as applied to aggressive space mapping. Microw. Opt. Technol. Lett..

[27] Akaike H. (1974). A new look at the statistical model identification. IEEE Trans. Autom. Control.

[28] HURVICH C.M., TSAI C.L. (1989). Regression and time series model selection in small samples. Biometrika.

[29] Brouwers H.J.H. (2008). Packing fraction of crystalline structures of binary hard spheres: A general equation and application to amorphization. Phys. Rev. E.

[30] Brouwers H.J.H. (2013). Random packing fraction of bimodal spheres: An analytical expression. Phys. Rev. E.

[31] Meng L., Lu P., Li S. (2014). Packing properties of binary mixtures in disordered sphere systems. Particuology.

[32] Shi Y., Zhang Y. (2008). Simulation of random packing of spherical particles with different size distributions. Appl. Phys. A.

[33] Nordström J., Welch K., Frenning G., Alderborn G. (2008). On the physical interpretation of the Kawakita and Adams parameters derived from confined compression of granular solids. Powder Technol..

[34] Mitra B., Hilden J., Litster J. (2018). Assessment of intragranular and extragranular fracture in the development of tablet tensile strength. J. Pharm. Sci..

[35] Mitra B., Hilden J., Litster J.D. (2016). Effects of the granule composition on the compaction behavior of deformable dry granules. Powder Technol..

[36] Nordström J., Alderborn G., Frenning G. (2018). Compressibility and tablet forming ability of bimodal granule mixtures: Experiments and DEM simulations. Int. J. Pharm..

[37] Johansson B., Alderborn G. (2001). The effect of shape and porosity on the compression behaviour and tablet forming ability of granular materials formed from microcrystalline cellulose. Eur. J. Pharm. Biopharm..

[38] Nordström J., Alderborn G. (2015). The Granule Porosity Controls the Loss of Compactibility for Both Dry- and Wet-Processed Cellulose Granules but at Different Rate. J. Pharm. Sci..

[39] Freitag F., Kleinebudde P. (2003). How do roll compaction/dry granulation affect the tableting behaviour of inorganic materials? Comparison of four magnesium carbonates. Eur. J. Pharm. Sci..

[40] Farber L., Hapgood K.P., Michaels J.N., Fu X.Y., Meyer R., Johnson M.A., Li F. (2008). Unified compaction curve model for tensile strength of tablets made by roller compaction and direct compression. Int. J. Pharm..

[41] Yohannes B., Gonzalez M., Abebe A., Sprockel O., Nikfar F., Kang S., Cuitino A. (2015). The role of fine particles on compaction and tensile strength of pharmaceutical powders. Powder Technol..

[42] Perez-Gandarillas L., Perez-Gago A., Mazor A., Kleinebudde P., Lecoq O., Michrafy A. (2016). Effect of roll-compaction and milling conditions on granules and tablet properties. Eur. J. Pharm. Biopharm..

[43] Kása P., Bajdik J., Zsigmond Z., Pintye-Hódi K. (2009). Study of the compaction behaviour and compressibility of binary mixtures of some pharmaceutical excipients during direct compression. Chem. Eng. Process. Process. Intensif..

[44] Ilić I., Kása P., Dreu R., Pintye-Hódi K., Srčič S. (2009). The compressibility and compactibility of different types of lactose. Drug Dev. Ind. Pharm..

[45] Sonnergaard J.M. (1999). A critical evaluation of the Heckel equation. Int. J. Pharm..

[46] Heckel R.W. (1961). Density-pressure relationships in powder compaction. Trans. Metal. Soc. AIME.

[47] Patel S., Kaushal A.M., Bansal A.K. (2006). Compression physics in the formulation development of tablets. Crit. Rev. Ther. Drug Carr. Syst..

